# ECG-Synchronized Computed Tomography in Assessing the Elastic Properties of the Ascending Aorta: Clinical and Experimental Study

**DOI:** 10.3390/diagnostics16050751

**Published:** 2026-03-03

**Authors:** Svetlana I. Sazonova, Viktor V. Saushkin, Dmitri S. Panfilov, Anatoliy B. Skosyrsky, Boris N. Kozlov

**Affiliations:** 1 Cardiology Research Institute, Branch of the Federal State Budgetary Scientific Institution “Tomsk National Research Medical Center of the Russian Academy of Sciences” (Cardiology Research Institute, Tomsk NRMC), 111a Kievskaya, 634012 Tomsk, Russia; saushkin@cardio-tomsk.ru (V.V.S.); pand2006@yandex.ru (D.S.P.);; 2 Laboratory of Structural and Functional Materials, Siberian Physical-Technical Institute (SPTI), National Research Tomsk State University (TSU), 36 Lenin Ave., 634050 Tomsk, Russia

**Keywords:** ascending aortic aneurysm, tensile testing, ECG-gated CT-angiography

## Abstract

**Background**: Recent studies have demonstrated the feasibility and potential of using ECG-synchronized computed tomography (CT) to assess the elastic and deformation properties of the aorta. However, to date, there is insufficient evidence to support the practical use of this approach. We aimed to study the association of CT-derived indices, characterizing ascending aorta elasticity, with the biomechanical properties of intraoperative ascending aorta (AsAo) samples, and to assess its predictive potential in non-surgical patients with ascending aorta dilatation. **Methods**: In total, 71 patients with AsAo dilatation (>45 mm) and 29 control patients (AsAo diameter < 40 mm) underwent ECG-synchronized CT-aortography. In 42 surgical patients, CT-derived parameters (circumferential strain, compliance, stiffness) were compared with the tensile strength and relative strain of intraoperative aortic samples. In 29 non-surgical patients (diameter 45–50 mm), the predictive potential of CT-derived elasticity indices was determined over 36 months of follow-up. **Results**: A moderate correlation was found between CT-derived strain/distensibility and ex vivo relative strain. CT data confirmed that dilated aortas are stiffer and less elastic than those in controls. In 29 non-surgical patients, CT elasticity parameters did not demonstrate the ability to predict adverse aneurysm progression. **Conclusions**: While CT can assess aortic elasticity correlated with ex vivo aortic properties, these parameters lacked prognostic value for the growth in small aneurysms.

## 1. Introduction

Thoracic aortic aneurysm (TAA) is a silent condition that can be complicated by vessel rupture or dissection and is associated with high mortality rates. The only way to prevent this catastrophe is to surgically replace the dilated segment. Currently, surgery is recommended for patients with non-heritable TAA when the maximum aortic diameter is ≥55 mm or when the aneurysm growth rate exceeds 0.5 cm/year [1]. However, recent studies have shown that a significant number of adverse aortic events occur in patients with an ascending aorta diameter of less than 55 mm [2,3]. Therefore, identifying new markers to improve risk stratification in patients with TAA and to optimize the choice between surgical and conservative management is highly relevant. In addition to diameter, other risk factors for aortic aneurysm rupture have been identified, such as ascending aortic length and the aortic arch angle [1]. Promising biomarkers for assessing aortic rupture risk include parameters of vessel stiffness and elasticity, which can be evaluated in vivo, using medical imaging techniques. Magnetic resonance imaging (MRI) is the most rapidly evolving technique in this field. It allows for effective assessment of both global (using pulse wave velocity) and local aortic stiffness (by measuring changes in vessel diameter during systole and diastole) [4]. Parameters derived from 4D flow MRI, such as flow displacement and eccentricity indices, are considered predictive biomarkers for aortic dilation and dissection, especially in asymptomatic individuals [4]. However, the use of MRI is limited by several factors, including availability, high cost, and a long acquisition time.

Computed tomography (CT) plays a key role in the diagnosis, risk stratification, and management of patients with aortic diseases [1]. Its main advantages, such as short image acquisition and processing times, good reproducibility, and wide availability [1], outweigh the disadvantages associated with radiation exposure and the administration of an iodine-based contrast agent. The method allows for obtaining detailed anatomical information, which directly impacts the quality of diagnostics, the accuracy of treatment planning, and the success of subsequent patient monitoring.

Moreover, recent studies have demonstrated the feasibility and potential of using ECG-synchronized (gated) CT to assess the elastic and deformation properties of the aorta [5]. Herein, the vessel deformation is assessed by measuring changes in diameter, cross-sectional area, and the length of longitudinal aortic segments between systole and diastole, followed by the calculation of indicators of stiffness, compliance, distensibility, and strain. There are ongoing efforts to enhance the precision of this method through the use of artificial intelligence [6,7]. However, to date, there is limited evidence to support the practical use of this approach.

Therefore, we aimed to study the association of CT-derived indices, characterizing ascending aorta (AsAo) elasticity, with the biomechanical properties of intraoperative AsAo samples, and to assess its predictive potential in patients with ascending aorta dilatation.

## 2. Materials and Methods

### 2.1. Study Population and Design

A prospective study included 71 patients with dilation of the ascending aorta greater than 45 mm, who were examined and followed at the clinics of our institution in 2020–2025. ECG-gated CT-aortography was performed for all patients.

The following inclusion criteria were defined:

• Age 45–70 years;
• Dilatation of the thoracic aorta greater than 45 mm as assessed by CT-aortography;
• Non-syndromic aortic diseases (idiopathic, familial);
• Preserved left ventricular (LV) function;
• Signed informed patient consent to participate in this study.

Exclusion criteria were as follows:

• Heart failure;
• History of myocardial infarction;
• History of stroke;
• Cardiac arrhythmias;
• Left ventricular dilatation (left ventricular end-diastolic diameter > 58 mm, left ventricular end-systolic diameter > 40 mm);
• Previous cardiac or thoracic aortic surgery;
• Arterial hypertension (resistant to pharmacotherapy);
• Congenital heart defects;
• Syndromic aortic diseases (e.g., Turner, Marfan, Ehlers–Danlos, Loeys–Dietz syndromes, and other genetic anomalies);
• Allergy to iodine-containing drugs;
• A refusal to participate in this study.

The patients were then divided into two groups based on the maximum systolic diameter of the ascending aorta and presence of indications for surgical treatment of aortic aneurysm:

Group 1 (*n* = 42): Patients with a maximum diameter of ascending aorta ≥50 mm with indications for surgical treatment of aortic aneurysm. All patients received ascending aortic replacement. The obtained intraoperative samples were subjected to tensile testing. The ex vivo tensile strength and strain of the ascending aorta were compared with CT data.

Group 2 (*n* = 29): Patients with a maximum diameter of ascending aorta of 45–49 mm without indications for surgical treatment of aortic aneurysm. This group of patients was used to evaluate the prognostic value of ascending aortic elasticity parameters derived from CT data. All patients of Group 2 were followed up prospectively in the outpatient clinic for 36 months after inclusion in this study. CT-aortography was performed at baseline, as well as at 12, 24 and 36 months of the follow-up period. The primary endpoints were an increase in the ascending aorta diameter by ≥50 mm; a thoracic aortic diameter growth rate of ≥3 mm/year based on CT data; aortic dissection or aortic rupture. The secondary endpoints were cardiovascular mortality and all-cause mortality.

Since reference values for CT-derived elasticity indices of the aorta have not yet been determined, a control group (*n* = 29) was included in this study. This group consisted of patients without aortic dilation who underwent CT coronary angiography to rule out obstructive atherosclerotic coronary artery disease. For these patients, the upper scan boundary during the contrast phase was set above the aortic arch.

Inclusion criteria for the patients in the control group were an age of 50–70 years, a maximum thoracic aortic diameter less than 40 mm (according to transthoracic echocardiography (TTE)), preserved left ventricular function, and informed consent to participate in this study.

Exclusion criteria for these patients were atherosclerotic coronary artery disease, heart failure, history of myocardial infarction, history of stroke, cardiac arrhythmias, a bicuspid aortic valve, congenital heart defects, prior cardiac or thoracic aortic surgery, diabetes mellitus, arterial hypertension (resistant to drug therapy), and refusal to participate in this study.

The CT-derived elasticity indices from the control group were compared with those of patients with ascending aortic dilatation. This was done to determine how aortic elasticity changes in patients who are not surgical candidates and to assess whether CT can detect these changes.

**Figure 1:**
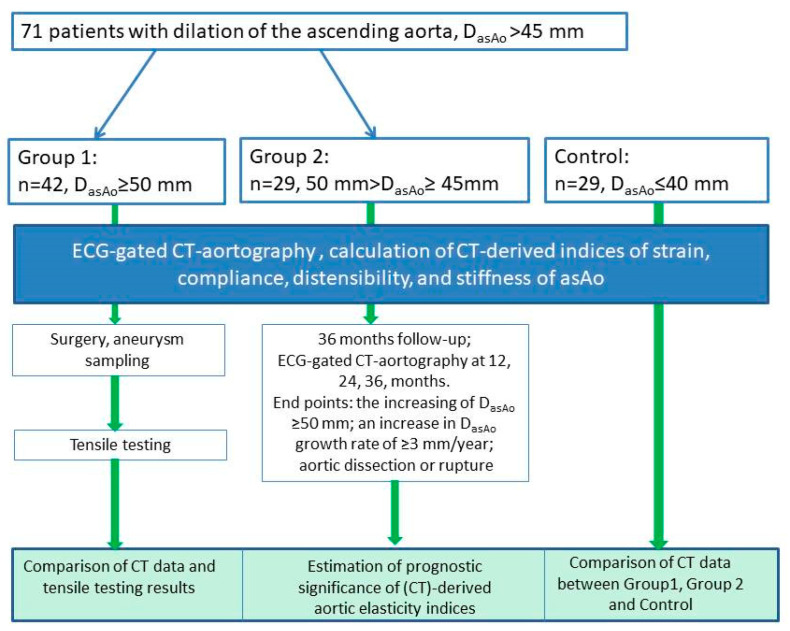
Study design. CT—computed tomography; D_asAo_—maximum diameter of ascending aorta.

The study design is shown in Figure 1. 

All the patients signed written consent to participate in this study. The study was approved by the Local Ethics Committee of the Research Institute of Cardiology at the Tomsk National Research Medical Center (Protocol No. 213 dated 12 May 2021) and was conducted in accordance with the ethical standards outlined in the 1975 Declaration of Helsinki.

### 2.2. CT-Aortography

To calculate elasticity indices and dimensions of the ascending aorta, all the patients underwent contrast-enhanced CT-aortography. The examination was performed on a Discovery NM/CT 570C hybrid cardiac computed tomography scanner (GE HealthCare, Haifa, Israel). For the contrast enhancement of the thoracic aorta, an iodine-containing infusion agent with an iodine concentration of 370–400 mg iodine/mL was used, in a volume of 60–110 mL (depending on the patient’s body weight), at a rate of 4–5.5 mL/s. Scan parameters: tube voltage 120 kV, tube current 300–600 mA with ECG modulation, gantry rotation speed 0.4 s, pitch: 0.20–0.22 (depending on heart rate). Images were reconstructed using standard protocols with a slice thickness of 0.625 mm. Processing of the obtained images was performed on an Advantage Workstations 4.3 (GE Healthcare) with subsequent measurement of the dimensions of all segments of the thoracic aorta.

The scanning protocol was based on retrospective ECG gating, which ensured continuous data acquisition throughout the entire cardiac cycle. Subsequent image reconstruction was performed at 10% increments of the R-R interval, generating 10 phases of the cardiac cycle.

For the measurement of morphometric parameters and calculation of strain indices, two representative phases were selected from the acquired dataset: end-systolic (40% or 50% R-R) and end-diastolic (80% or 90% R-R). The specific phase was chosen individually for each patient based on expert assessment of the image quality. The key selection criteria were the absence of dynamic artifacts (“blurring” of contours), the absence of spatial displacement of the aorta at the studied levels, and the presence of clear, verifiable borders of the aortic wall. This approach minimized measurement error and ensured accuracy in the calculation of biomechanical indices.

**Figure 2:**
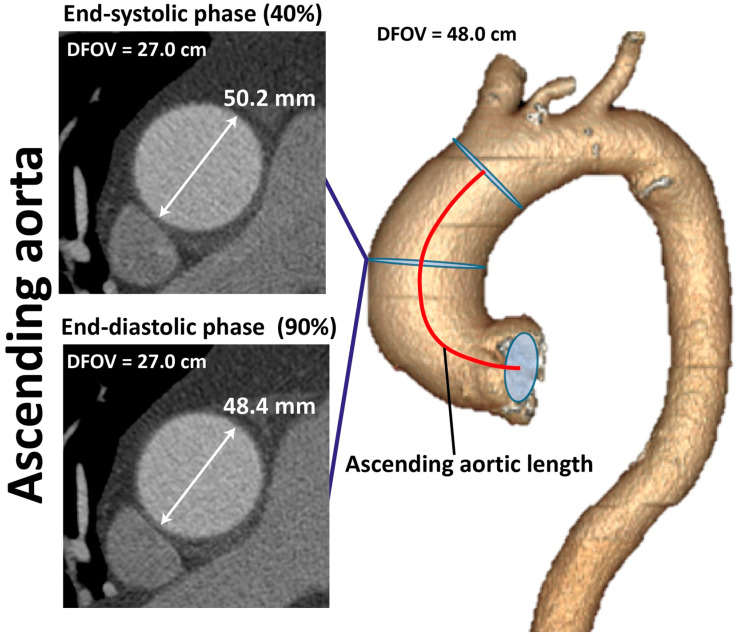
Morphometric processing of ascending aorta. DFOV—Display Field of View.

Morphometric processing was performed at the level of the ascending aorta, where the maximum diameter in systole and diastole was measured (Figure 2). The circumferential deformation, wall compliance, distensibility, and stiffness of the ascending aorta were calculated from these parameters using formulas 1–4 [7]. The AsAo length (the segment from the aortic annulus to the brachiocephalic trunk) was also measured (Figure 2).

(1) Circular strain (%) = ((Ds − Dd)/Dd) × 100%,

(2) Compliance (mm^2^/mmHg) = (π × (Ds^2^ − Dd^2^))/(4 × PP),

(3) Distensibility (%/mmHg) = (100 × (Ds^2^ − Dd^2^))/(PP × Dd^2^),

(4) Stiffness index (β) = (ln (SBP/DBP) × Dd)/(Ds − Dd),

where Ds—maximal diameter of ascending aorta in systole; Dd—maximal diameter of ascending aorta in diastole; PP—pulse pressure; SBP—systolic blood pressure; DBP—diastolic blood pressure.

### 2.3. Sample Preparation and Biomechanical Testing (For Group 1)

**Figure 3:**
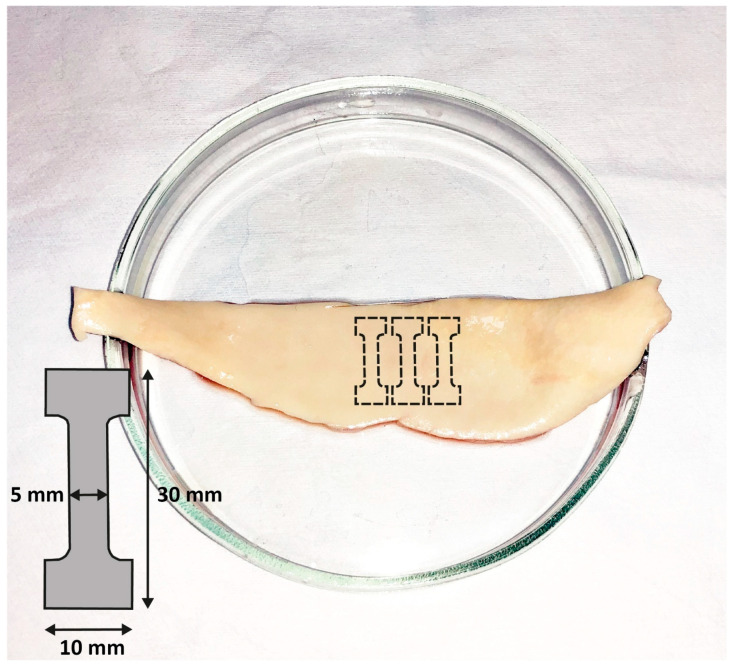
Excision method for aortic aneurysm specimens for tensile testing. Icon indicated by a dash-dot line shows the shape of the excised aortic specimen.

Sample preparation and biomechanical testing were performed in accordance with the techniques described earlier [8,9]. Immediately after aneurysm resection, the excised aortic wall (Figure 3) was placed in a cooled (+4 °C) Krebs–Henseleit solution (Sigma-Aldrich, Merck Life Science LLC, Moscow, Russia), and then within 2 h, a vessel fragment was cut with a scalpel along the lesser curvature. Three specimens were then cut in the circumferential directions using a 3 × 1 cm stamp (Figure 3).

**Figure 4:**
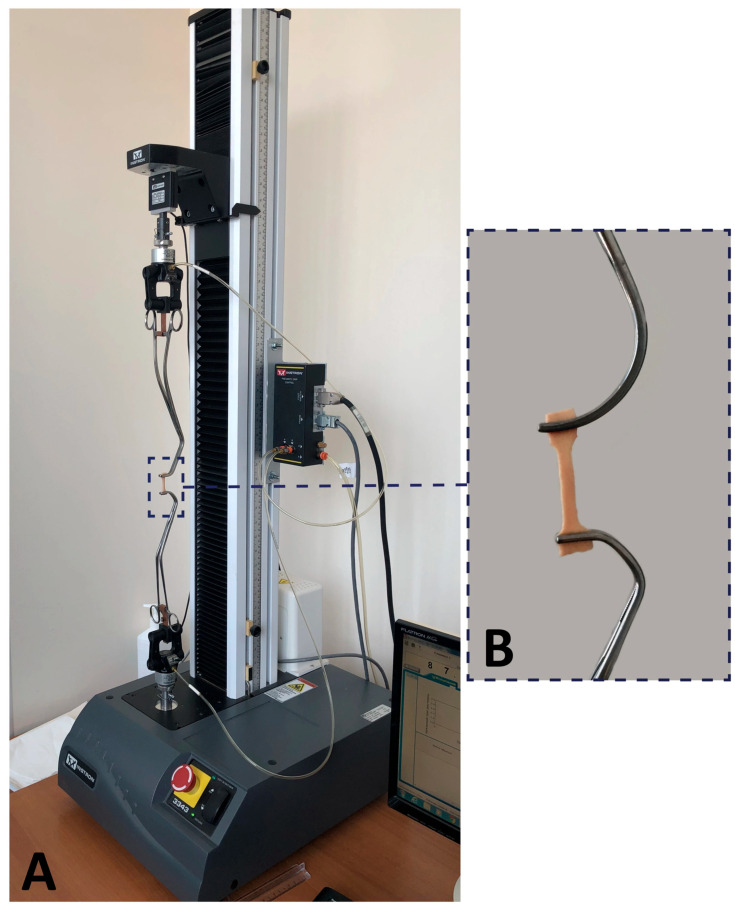
Tensile testing of aortic sample. (**A**) Instron 3343 testing machine with an aortic specimen attached. (**B**) Specimen of ascending aorta, clamped in atraumatic grips.

Each sample was mounted in atraumatic vascular clamps, which were securely fixed to the standard grips of the testing machine (Figure 4), and which were stretched to break at a rate of 1 mm/min.

Testing parameters are presented in Table 1.

**Figure 5:**
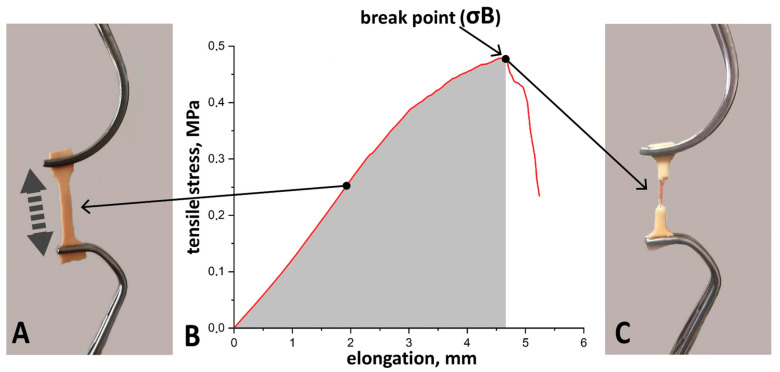
Ex vivo uniaxial tensile testing of an ascending aorta specimen. (**A**) Ascending aorta specimen during uniaxial tensile loading. (**B**) Typical load–extension curve (red line) for a circular specimen of ascending aorta. (**C**) Ascending aorta specimen after rupture. Arrows indicate the peak of the curve, corresponding to the ultimate tensile strength (σB, MPa) and the break point.

For each sample, tensile strength (σB, MPa) was defined as the peak value on the load–extension curve (Figure 5). The relative strain (ε, mm/mm × %) was calculated as the percentage elongation at failure. Mean values for all parameters were then determined for all samples.

### 2.4. Statistical Analyses

Statistical data processing was performed using Statistica 10 software (StatSoft, Inc., Tulsa, OK, USA). The normality of the variables’ distribution was tested with the Shapiro–Wilk test. Since the normal distribution was not confirmed for all the quantitative data, the results were presented as a median (Me) and quartiles (Q1; Q3). Statistical significance of quantitative data was determined using the non-parametric Mann–Whitney U-test. Correlation analysis was performed with the Spearman correlation test. To evaluate the independent predictors of rapid aneurysm growth, forward-stepwise logistic regression analysis was used with an entry criterion of *p* < 0.05 and a removal criterion of *p* < 0.1. The receiver operating characteristic (ROC) curve analysis was performed to evaluate the sensitivity and specificity of CT indexes to predict unfavorable aneurysm progression. A *p* < 0.05 indicated a statistically significant difference.

## 3. Results

### 3.1. Comparisons of Group 1 and Control Group

In Group 1, all 42 patients (100%) underwent hemiarch replacement. Of these, 16 (38.1%) patients had concomitant aortic valve replacement and 8 (19%) patients received aortic valve repair.

Baseline characteristics of patients of Group 1 and their comparison with the control group are presented in Table 2. The majority of Group 1 patients (72.8%) had a tricuspid valve, and more than a half of the patients suffered from arterial hypertension. The group consisted predominantly of males. No significant differences were observed in the clinical characteristics between Group 1 and the control group.

At the same time, compared to the control group, Group 1 showed the expected decrease in circumferential strain and increase in stiffness of the ascending aortic wall (Table 3). We also obtained a contradictory finding—an increase in compliance alongside an increase in stiffness.

### 3.2. Comparison of CT-Derived Indices of Ascending Aorta Elasticity with the Biomechanical Properties of Intraoperative Ascending Aorta Samples

CT-derived aortic elasticity indices, tensile strength, relative strain and their correlations are presented in Table 4. According to the statistical analysis, tensile strength did not correlate either with aortic deformation indices derived from CT data or with the maximum diameter. At the same time, we revealed a moderate direct correlation between relative strain (ε, mm/mm × %) and both CT-derived circumferential strain (%) and distensibility (%/mmHg), and a negative correlation with stiffness.

### 3.3. Comparisons of Group 2 Characteristics with the Control Group

The clinical characteristics of patients of Group 2 and the results of their comparison with the control group are presented in Table 5. The groups were similar with regard to clinical and demographic characteristics, except for the dimensions of the ascending aorta (diameter and length), which were predetermined by the inclusion criteria.

Similarly to Group 1, it was found that in Group 2, a significantly lower circumferential strain value and higher stiffness and compliance values were detected in patients with ascending aortic aneurysm when compared with the control patients (Table 6).

### 3.4. Results of the Follow-Up in Group 2

During the follow-up period, no adverse aortic complications (secondary endpoints) were registered among the patients of Group 2. The first control examination (at 12 months after inclusion in this study) was completed by all 29 (100%) patients, the second (at 24 months) by 10 patients (34.5%), and the third (at 36 months) by 6 patients (20.7%).

At 12 months, the primary endpoint was reached by 19 (65.5%) patients. Among them, 13 patients (45%) had an increase in aortic diameter of greater than 50 mm, and 6 patients (21%) demonstrated a rate of ascending aortic diameter growth of more than 3 mm/year.

By the 24-month follow-up, the primary endpoint was also reached by four more patients: in three patients, the aortic diameter exceeded the surgical threshold, and one patient showed an accelerated aneurysm growth rate.

Thus, 23 of 29 (79.3%) patients of Group 2 reached the primary endpoint within 36 months of follow-up. Since the number of patients who had not reached the endpoints after 3 years of follow-up was insufficient for statistical analysis, we subsequently assessed predictors of rapid progression of ascending aortic aneurysm within the first year of the follow-up. For this, we divided the study population into two subgroups, which included those with “negative dynamics” of aortic aneurysm and those “without dynamics”, and compared their CT parameters (Table 7).

According to our results, the maximum diameter of the ascending aorta was the only parameter that significantly differed between the subgroups. All other measured indices, including the CT-derived elasticity parameters, did not differ significantly.

Univariable logistic analysis and a subsequent multivariable analysis, which, in addition to the aortic diameter, included clinical features and AsAo length, demonstrated that only the diameter was significantly associated with rapid aneurysm progression (Table 8).

**Figure 6:**
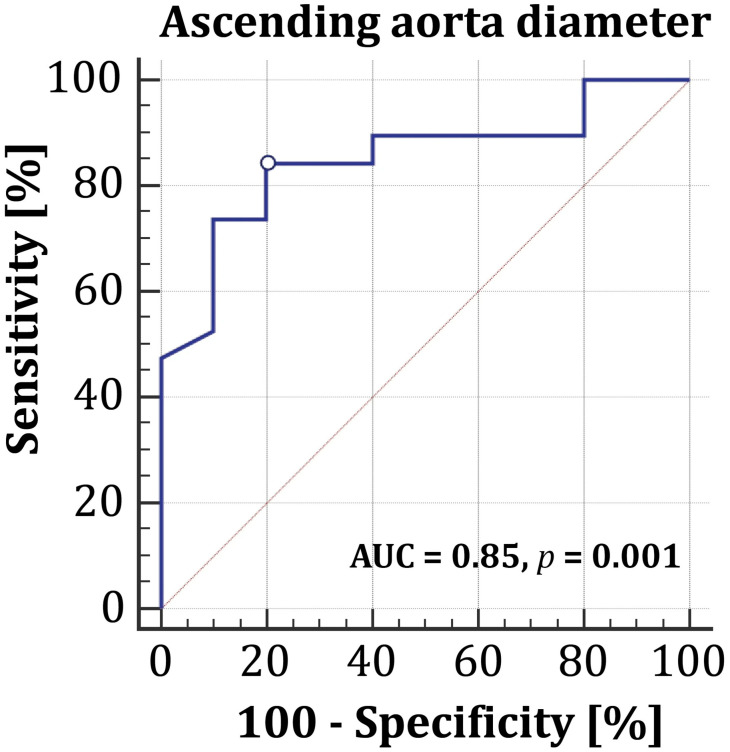
Receiver operating curve for ascending aorta diameter (cut-off point 47.6 mm) in predicting rapid aneurysm progression in patients without indications for surgery.

Subsequent ROC curve analysis (Figure 6) showed that an ascending aorta diameter ≥ 47.6 mm (cut-off point) is associated with a rapid aneurysm growth rate (sensitivity 84.2%, specificity 80.0%, AUC = 0.85, *p* = 0.001).

## 4. Discussion

The major findings of the present study are that CT-derived indices of ascending aorta elasticity correlate with relative strain, defined ex vivo, and that, according to CT data, the dilated ascending aorta is stiffer and less elastic than the normal ascending aorta. However, in patients with a dilated ascending aorta without indications for surgery, CT-derived elasticity parameters were not significant predictors of rapid aneurysm progression. In our study, the only independent predictor of aneurysm progression was the maximum diameter of the ascending aorta, with a cut-off point ≥ 47.6 mm. This finding reignites the discussion on decreasing the surgical threshold for the AsAo diameter.

The issue of choosing between surgical and therapeutic strategies remains relevant for patients with small thoracic aortic aneurysms, when the vessel diameter is less than 55 mm. It has been shown that the risk of aortic wall rupture in this category of patients is 2%, which is slightly higher than that (1.8%) in patients with an aortic diameter greater than 55 mm [10,11]. Furthermore, a composite endpoint, including aortic rupture, dissection, and death, is only slightly lower in the dilatation group compared to the aneurysm group (4.2% vs. 5.8%) [10,11]. According to a meta-analysis by Henry M. et al. [12], non-syndromic aneurysms of the ascending thoracic aorta generally grow at a rate of approximately 0.3–0.5 mm per year. Similar results were obtained in a recent study by Obel L.M. et al. [13], which, in a large cohort of patients (*n* = 2195), demonstrated very slow growth of non-syndromic thoracic aortic aneurysms less than 50 mm, over 5 years. Despite this, in the aforementioned study, the majority of adverse aortic events (95%) occurred in patients with a pre-event aortic diameter of less than 50 mm, highlighting the need for individualized risk stratification in addition to the diameter [13]. The authors of both papers are of the same opinion that annual examinations of patients of this category are not advisable, and it is acceptable to extend the interval between aortic imaging. The results of our study differ from those discussed above. In particular, of the 29 patients included in this study, the surgical threshold was reached by 65.5% already in the first year, and by 79% of patients by the end of the third year of the follow-up, highlighting the necessity of annual imaging surveillance to monitor aneurysm size. The differences in results, aside from the significantly smaller sample size, may be due to clinical characteristics of the patients, population features, and adherence to antihypertensive therapy. However, these issues require further investigation.

The influence of patients’ clinical characteristics, as well as the hemodynamic and biomechanical parameters of the aorta, on the rate of aneurysm growth remains poorly studied. It is generally considered that the biomechanical characteristics of the aorta, particularly those assessed using medical imaging techniques, are a promising tool for personalized risk stratification in patients with ascending aortic aneurysm. At the same time, over the past 10 years, researchers have repeatedly emphasized the need for large-scale validation of these indicators in comparison with aortic biomechanics indices, estimated ex vivo [5]. Despite this, the number of published studies, comparing in vivo and ex vivo elastic parameters is insufficient.

Initially, Pasta et al. [14] demonstrated the feasibility of using ECG-gated CT to assess strain across all aortic segments in 25 TAA and 7 non-TAA patients. Subsequently, de Beaufort et al., in a study of 10 patients with abdominal or thoracic aortic aneurysms, showed that ECG-gated CT allows the evaluation of not only circumferential strain but also longitudinal pulsatile deformation of the ascending aorta, with significant interindividual variations in these parameters [15].

Subsequently, Farzaneh et al. [16] in a small sample consisting of 11 patients with ascending aortic aneurysm demonstrated a close correlation between the CT-derived measure of local stiffness of the ascending aorta and the biomechanical characteristics of intraoperative specimens.

In a recent study by Yokota et al. [17], which included 49 patients undergoing ascending aortic surgery, a close correlation was demonstrated between the elastic modulus, which was calculated from ECG-gated CT images using a theoretical formula, and the results of ex vivo loading tests on resected aortic specimens. These results support the potential of ECG-gated CT-based aorta elasticity assessment for risk stratification of aortic catastrophes.

The results of our study are in line with the literature, since a direct moderate correlation between ex vivo relative strain and CT-derived circumferential strain was revealed. At the same time, the strength of the correlation between in vivo and ex vivo elasticity parameters could have been negatively influenced by several factors. First, the process of the aortic samples isolating may lead to changes in their behavior, affecting the results of ex vivo tests. Second, aortic tissue is anisotropic and stretched biaxially in vivo. Therefore, uniaxial testing may not have fully reflected the actual biomechanical properties of the aortic wall. Biaxial testing is considered to be more representative of physiological conditions, but this method also has certain limitations [18].

A somewhat larger number of published studies are devoted to the comparison of MRI-derived biomechanical parameters of the aorta with reference-standard mechanical testing of TAA specimens from surgical repairs [19]. All of them were performed on samples of less than 100 patients. Particularly, it was shown that wall share stress correlated with a reduced aneurysm strength, measured in vitro [20,21]. In an experimental study by Dong et al. [22], it was demonstrated that MRI-derived aorta stiffness correlated with histopathological analyses, and inversely correlated with wall strength, estimated in ex vivo uniaxial tensile and burst testing. The use of computer modeling and artificial intelligence also allows for obtaining elasticity parameters, which are closely associated with ex vivo data [23,24].

However, despite the fact that the majority of studies indicate a close association between elasticity indices, determined by medical imaging, and biomechanical parameters of intraoperative aortic samples, the prognostic value of aortic elasticity estimated in vivo is not still clear. In the presented study, CT-derived aortic elasticity parameters did not show prognostic significance for rapid aneurysm growth in patients with small aneurysms, although they differed from the control group. However, due to the small sample size in the subgroup of patients without indications for surgery and the number of patients who had not reached the study endpoints, the statistical power of the analysis was limited, and the findings should therefore be considered preliminary. Furthermore, no patients with aortic complications were observed during the follow-up period in our study. Therefore, based on the data obtained, it is premature to draw definitive conclusions regarding the true prognostic value of CT-derived elasticity parameters in patients with ascending aorta aneurism. It is also possible that the dynamics of changes in vascular stiffness are of greater clinical significance. Thus, the study by Zamirpour et al. [25] emphasizes that in a population of non-surgical patients, large temporal changes in peak circumferential stress, which was calculated using finite element analysis of ECG-gated CT aorta scans, were associated with all-cause mortality. However, the above method of wall stress estimation has not been translated into clinical practice yet.

When discussing the clinical application of aortic elasticity parameters, it is important to emphasize that their age-specific reference values have not yet been established. Therefore, our study utilized a control group consisting of age-matched patients without aortic pathology. Using these data, we demonstrated that aortic stiffness increases significantly even at vessel diameters under 50 mm, indicating morphological remodeling in the wall of the ascending aorta and an increased risk of aortic dissection or rupture in these patients. At the same time, a considerable individual variability in CT-derived aortic elasticity parameters was observed. Thus, we propose that their dynamic assessment will have greater clinical utility than a simple comparison with static reference values.

The present study has several limitations, the most notable of which is the relatively small sample size, especially for patients, who did not reach the study endpoints during the follow-up. Moreover, we used a uniaxial tensile testing method, which may not fully replicate in vivo three-dimensional conditions. A paradoxical result was also observed, with an increase in compliance occurring concomitantly with increased stiffness. This result is evidently attributed to the dominant geometric effect of a larger baseline diameter, which is squared in the calculation formula (2) [7] and outweighs the reduction in intrinsic wall elasticity. Consequently, compliance values derived from CT using the formula above cannot be used to compare groups of patients with significantly different ascending aortic diameters, although a longitudinal (or intragroup) dynamic assessment of compliance is possible. We believe that formula (2) requires refinement—for example, through the introduction of correction factors—to improve the accuracy of aortic compliance calculations based on CT data.

## 5. Conclusions

The present study demonstrated a correlation between CT-derived elasticity of the ascending aorta and ex vivo relative strain. It also confirms, via CT data, that dilated aortas are stiffer and less elastic than normal ones. At the same time, CT-derived elasticity parameters did not demonstrate the ability to predict rapid progression of ascending aortic aneurysm in patients without indications for surgery. To determine the clinical significance of aortic elasticity characteristics, it is important to perform larger controlled studies, establishing reference values and determining the prognostic significance of the indicators themselves, or the dynamics of their changes.

## Figures and Tables

**Table 1:** Testing parameters.

Parameter	Measuring Range	Value Determination Accuracy
Tension, N	0–50	±0.5%
Traverse travel speed, mm/min	0.005–500	±0.2%
Linear resizing, mm:		0.05%
Stretching	15–1000	
Contraction	0–1000	

**Table 2:** Baseline characteristics of patients of Group 1 vs. control group.

Parameter	Parameter		
	Group 1 (*n* = 42)	Control (*n* = 29)	*p*-Value
Age, years *	62 [54; 67]	60 [52; 67]	0.181
Males (*n* (%))	28 (66.7)	18 (62.1)	0.476
Diabetes mellitus (*n* (%))	3 (7.1)	2 (6.9)	0.715
Systolic blood pressure, mm Hg *	130 [120.0; 143.0]	130 [120; 140]	0.681
Diastolic blood pressure, mm Hg *	90 [80.0; 90.0]	84 [80; 90]	0.961
Arterial hypertension *n* (%)	26 (61.9)	13 (44.8)	0.413
Dyslipidemia (*n* (%))	11 (26.2)	6 (20.7)	0.383
Tricuspid aortic valve (*n* (%))	31 (73.8)	26 (89.7)	0.039
Bicuspid aortic valve (*n* (%))	11 (26.2)	3 (10.3)	0.039

Footnotes: *—Data are presented as Me [Q1; Q3].

**Table 3:** Elasticity parameters of the ascending aortic wall in Group 1 vs. control group.

Parameter	Group 1 *n* = 42	Control *n* = 29	*p*-Value
Circumferential strain (%)	2.75 [0.60; 5.12]	5.45 [2.20; 10.70]	0.002
Compliance (mm^2^/mmHg)	2.25 [0.59; 4.16]	1.06 [1.18; 2.04]	0.007
Distensibility (%/mmHg)	0.13 [0.03; 0.22]	0.12 [0.02; 0.29]	0.200
Stiffness index (β)	13.94 [8.06; 29.75]	10.54 [3.51; 17.69]	0.017

Footnotes: Data are presented as Me [Q1; Q3].

**Table 4:** CT data and their correlation with biomechanical parameters of ascending aorta samples.

Parameter	Value *	Tensile Strength (σB, MPa)	Relative Strain (ε, mm/mm × %)
		0.36 [0.2; 0.52] *	8.1 [6.58; 9.06] *
Circumferential strain (%)	2.76 [0.94; 5.19]	-	r = 0.3 (*p* < 0.05)
Compliance (mm^2^/mmHg)	2.25 [0.59; 4.16]	-	-
Distensibility (%/mmHg)	0.13 [0.03; 0.22]	-	r = 0.32 (*p* < 0.05)
Stiffness index (β)	13.9 [8.06; 29.75]	-	r = −0.32 (*p* < 0.05)
Maximum diameter (mm)	50.3 [50.0; 52.7]	-	-

Footnotes: *—Data are presented as Me [Q1; Q3]; “-”—no significant correlations; r—Spearman’s rank correlation coefficient.

**Table 5:** Clinical and instrumental characteristics of patients of Group 2 and control group.

Parameter	Group 2 *n* = 29	Control *n* = 29	*p*-Value
Age, years *	66 [57; 70]	60 [52; 67]	0.181
Males (*n* (%))	20 (69.0)	18 (62.1)	0.701
Systolic blood pressure, mm Hg *	130 [110; 170]	130 [120; 140]	0.681
Diastolic blood pressure, mm Hg *	85 [80; 90]	84 [80; 90]	0.961
Arterial hypertension (*n* (%))	16 (55.2)	13 (44.8)	0.413
Dyslipidemia (*n* (%))	5 (17.2)	6 (20.7)	0.383
Diabetes mellitus (*n* (%))	2 (6.9)	2 (6.9)	0.715
Tricuspid aortic valve (*n* (%))	23 (79.3)	26 (89.7)	0.128
Bicuspid aortic valve (*n* (%))	6 (20.7)	3 (10.3)	0.128
Maximum diameter (mm) *	49.1 [48.6; 49.4]	35.2 [32.2; 37.8]	0.001
Ascending aorta length (mm) *	108.8 [103.1; 120.0]	83.7 [74.7; 89.1]	0.001

Footnotes: *—Data are presented as Me [Q1; Q3].

**Table 6:** Elasticity parameters of the ascending aortic wall in Group 2 vs. control group.

Parameter	Group 2 *n* = 29	Control *n* = 29	*p*-Value
Circumferential strain (%)	2.49 [1.24; 5.22]	5.45 [2.20; 10.70]	0.021
Compliance (mm^2^/mmHg)	2.44 [1.45; 3.75]	1.06 [1.18; 2.04]	0.001
Distensibility (%/mmHg)	0.14 [0.08; 0.21]	0.12 [0.02; 0.29]	0.733
Stiffness index (β)	13.35 [7.76; 43.49]	10.54 [3.51; 17.69]	0.032

Footnotes: Data are presented as Me [Q1; Q3].

**Table 7:** Comparison of CT-derived morphometric and elasticity parameters of the ascending aorta in patients with rapid aneurysm progression vs. patients without aneurysm growth dynamics.

Parameter	Aneurysm Progression *n* = 19	No Progression *n* = 10	*p*-Value
Maximum diameter (mm)	48.3 [46.7; 49.6]	46.0 [45.2; 47.4]	0.029
Circumferential strain (%)	2.62 [1.95; 4.93]	2.45 [1.38; 4.67]	0.405
Compliance (mm^2^/mmHg)	2.47 [1.41; 3.80]	1.52 [1.20; 3.18]	0.317
Distensibility (%/mmHg)	0.13 [0.07; 0.21]	0.110 [0.07; 0.19]	0.642
Stiffness index (β)	8.03 [7.05; 17.08]	13.21 [7.09; 23.75]	0.716
Length (mm)	109.35 [103.8; 118.6]	107.50 [100.30; 118.70]	0.840

Footnotes: Data are presented as Me [Q1; Q3].

**Table 8:** Univariate and multivariate logistic regression analysis for the first endpoint.

Parameter	Univariate Regression Analysis OR (95% Cl)	*p*-Value	Multivariate Regression Analysis OR (95% Cl)	*p*-Value
Male sex	0.75 (0.14–3.90)	0.732		Ns
Age	0.97 (0.9–1.02)	0.447		Ns
Arterial hypertension	0.30 (0.06–1.52)	0.145		Ns
Atherosclerosis	0.87 (0.29–2.57)	0.801		Ns
Dyslipidemia	0.10 (0.01–1.09)	0.059		Ns
Diabetes mellitus	0.58 (0.03–10.47)	0.102		Ns
Tricuspid aortic valve	3.85 (0.38–38.35)	0.251		Ns
Maximum diameter (mm)	1.61 (1.06–2.43)	0.023 *	1.54 (1.02–2.35)	0.042 #
Circumferential strain (%)	0.89 (0.71–1.12)	0.397		Ns
Compliance (mm^2^/mmHg)	1.28 (0.89–1.85)	0.180		Ns
Distensibility (%/mmHg)	2.79 (0.55–14.17)	0.217		Ns
Stiffness index (β)	0.99 (0.97–1.01)	0.374		Ns
Length (mm)	1.06 (0.99–1.14)	0.082		Ns

Footnotes: *—significance in univariate analysis; #—significance in multivariate analysis; Ns—not significant.

## Data Availability

The data presented in this study are available on request from the corresponding author due to institutional policies.

## Abbreviations

AsAo	Ascending aorta
CT	Computed tomography
TAA	Thoracic aortic aneurysm
MRI	Magnetic resonance imaging
LV	Left ventricular
TTE	Transthoracic echocardiography
ROC	Receiver operating characteristic
AUC	Area under curve
σB	Tensile strength
ε	Relative strain
